# Frictional Wear Behavior of Water-Lubrication Resin Matrix Composites under Low Speed and Heavy Load Conditions

**DOI:** 10.3390/polym16192753

**Published:** 2024-09-29

**Authors:** Wu Ouyang, Feipeng Pan, Lei Wang, Ruicong Zheng

**Affiliations:** 1 State Key Laboratory of Maritime Technology and Safety, Wuhan University of Technology, Wuhan 430063, China; ouyangw@whut.edu.cn; 2 School of Transportation and Logistics Engineering, Wuhan University of Technology, Wuhan 430063, China; 333112@whut.edu.cn; 3 Reliability Engineering Institute, National Engineering Research Center for Water Transport Safety, Wuhan 430063, China; 4 East Lake Laboratory, Wuhan 420202, China; 5 China Ship Development and Design Center, Wuhan 430064, China; wanglei626@126.com

**Keywords:** wear behavior, water-lubricated bearing, resin matrix composite, low speed and heavy load

## Abstract

Resin matrix composites are commonly utilized in water-lubricated stern tube bearings for warship propulsion systems. Low-speed and high-load conditions are significant factors influencing the tribological properties of stern tube bearings. The wear characteristics of resin-based laminated composites (RLCs), resin-based winding composites (RWCs), and resin-based homogeneous polymer (RHP) blocks were investigated under simulated environmental conditions using a ring-on-block wear tester. Simulated seawater was prepared by combining sodium chloride with distilled water. The wetting angle, coefficient of friction (COF), and mass loss were measured and compared. Additionally, their surface morphologies were examined. The results indicate a significant increase in the COFs for the three materials with an increased speed or load under dry conditions. The COF of the RLCs is the lowest, indicating that it has superior self-lubricating properties. In wet conditions, the COFs of the three materials decrease with an increasing speed or load, exhibiting a pronounced hydrodynamic effect. The COF and mass loss of RWCs are the highest, while RLCs and RHP exhibit lower COFs and mass loss values. The reticulated texture and flocculent fibers on the surface of RLC enhance the heat diffusion and improve the material wettability and water storage capacity. The surface of RWC is dense, and the friction area under dry conditions is melted and brightened. The surface of RHP is smooth, while the worn material forms an agglomerate and exhibits susceptibility to burning and blackening under dry conditions. The laminated formation method demonstrates superior tribological performance throughout the wear evolution process.

## 1. Introduction

Water-lubricated bearings have been used in marine stern tube bearings for over 170 years. Compared with oil-lubricated bearings, water-lubricated bearings have resource-saving characteristics, environmental friendliness, and noise reduction. They meet the development needs of energy saving, environmental protection, and green shipping. Water lubrication has become the main form of naval vessel stern tube bearings and is widely used in civil ships [1]. The water-lubricated bearing condition of the ship is special. On the one hand, low-viscosity seawater greatly reduces the bearing capacity [2]. On the other hand, as the ship tonnage increases, the propeller cantilever results in a serious edge load in the water-lubricated bearing [3]. In addition, when the rotation speed is low, the bearing makes it difficult to form a hydrodynamic water film at 10 rotations/min. Under harsh conditions, water-lubricated bearings often suffer from wear, spalling, delamination, and noise [4,5].

Improving the material is one of the key methods to improve the performance of water-lubricated bearings [6,7]. With the advancement of materials technology, developed bearing materials have included natural ironwood, birch laminate, fabric laminate, natural rubber (NR), and synthetic nitrile rubber (NBR). With higher anti-wear and -noise performance requirements in modern ships, traditional single rubber has insurmountable shortcomings. Modified rubber [8,9] and resin matrix composites have gradually appeared [10,11].

Resin matrix composites are widely used in water-lubricated bearing materials. Examples include Thordon [12], Feroform [13], fiber-reinforced phenolic resin, polyamide, ultrahigh-molecular-weight polyethylene (UHMWPE) [14], etc. Many experiments on the tribological properties of different materials under simulated environments have been proposed. P De Baets [15] compared the tribological properties of six bearing materials in seawater. It was found that polyamide has a very small wear rate and a small coefficient of friction (COF) at the same load and speed. Glass fiber (GF) and/or carbon fiber (CF) filler materials can improve the friction and wear characteristics of UHMWPE-based composites under dry and water-lubricated conditions [16]. The research found that counterface morphology greatly influences composites’ friction and wear characteristics [17]. Because of the corrosion of the counterface by seawater [18], the wear rate of the composite against GCr15 under the lubrication of seawater was much larger than that under other conditions [19]. Tribological tests of polyether ether ketone (PEEK) and PEEK containing 30 wt.% glass fiber against AISI D2 steel were carried out [20]. The results show that the friction coefficient of composites decreased with an increase in normal pressure at relatively low loads. A similar phenomenon was proposed by another researcher [21]. The forming process of materials is also an important factor. The weave density has an important influence on the tribological behavior of hybrid PTFE/Kevlar fabric composites. With an increasing weave density, the wear rate increases, and the friction coefficient decreases [22]. Roughness is another factor. Golchin et al. [23] found that composites with a lower surface roughness had a lower dynamic friction. N. Liu et al. [24] observed abrasion patterns perpendicular to the sliding direction on the worn surface of composites under seawater lubrication or dry conditions. It can be seen that there are many factors affecting the tribological properties of resin matrix composites, and the influence mechanism of the forming process remains to be revealed.

This study investigated the effects of the manufacturing process and operating conditions (low speed, heavy load, and seawater) on the tribological properties of resin matrix composite stern bearings. Details of the experimental apparatus are explained in Section 2. The results are presented in Section 3, followed by discussions and conclusions in Section 3 and Section 4, respectively.

## 2. Methods and Experiments

### 2.1. Experimental Materials

There are many forming processes of resin matrix composites, such as filament winding [25], extrusion forming [25], laminating [26], and injection forming [25]. Materials for three representative forming processes were selected, including resin-based laminated composite (RLC), resin-based winding composite (RWC), and resin-based homogeneous polymer (RHP).

The primary raw materials, epoxy resin and polyhexamethylene adipamide, were obtained from Guangzhou Zhonggao Chemical Co. (Guangzhou, China), while polyurethane was sourced from Fujian Huide New Material Co. (Fujian, China). Additionally, fibers were acquired from Weihai Guangwei Composite Material Co. (Weihai, China) and fillers purchased from Shanghai Aladdin Biochemical Technology Co. (Shanghai, China).

The RLC comprised 64.6 wt.% polyhexamethylene adipamide and epoxy resin combined with 7 wt.% CaCO_3_ as a filler. Polyester and aramid fibers were used for reinforcement, while solid lubricants including graphite, MoS_2_, and polytetrafluoroethylene (PTFE) accounted for a total of 22 wt.%. During preparation, the matrix material, reinforcing phase, and filler were blended to produce prepregs, which were subsequently layered into laminates with alternating orientations of 0° and 90°. The fiber prepregs were positioned in a mold, integrated into a pressing unit, and subjected to hot pressing. The mold was hot-pressed at 200 °C and 10 MPa for 1 h, followed by cold pressing at ambient temperature under the same pressure for an additional hour before demolding to obtain the samples. Due to limitations in the hot-pressing process, the edges of the molded specimens were irregular; therefore, the test specimens were milled and machined to ensure uniformity. In comparison, the RWC was formulated with 43.6 wt.% epoxy resin, also incorporating 7 wt.% CaCO_3_. A variety of fibers, including cotton, aramid, carbon, glass, and polyacrylonitrile fibers, served as reinforcements, while graphite and MgCa(CO_3_)_2_ were used as the solid lubricants, totaling 54 wt.%. The fibers were initially grouped into bundles and immersed in a resin mixture containing epoxy resin, filler, and solid lubricants. The prepreg was wound onto a rotating mandrel under controlled tension facilitated by a tension controller. The material underwent curing at 95 °C for 1 h, followed by additional curing at 135 °C for 3 h. The resulting prepregs were then processed into specimens for testing. The RHP consisted of a homogeneous polyurethane material. Initially, the polyurethane particles were dried in a vacuum oven at 50 °C for 3 h. The samples were then closed-molded using a syringe machine at 200 °C for 15 s, followed by demolding and cooling for 30 s. After resting at room temperature for 12 h, the samples were subjected to friction tests.

**Figure 1:**
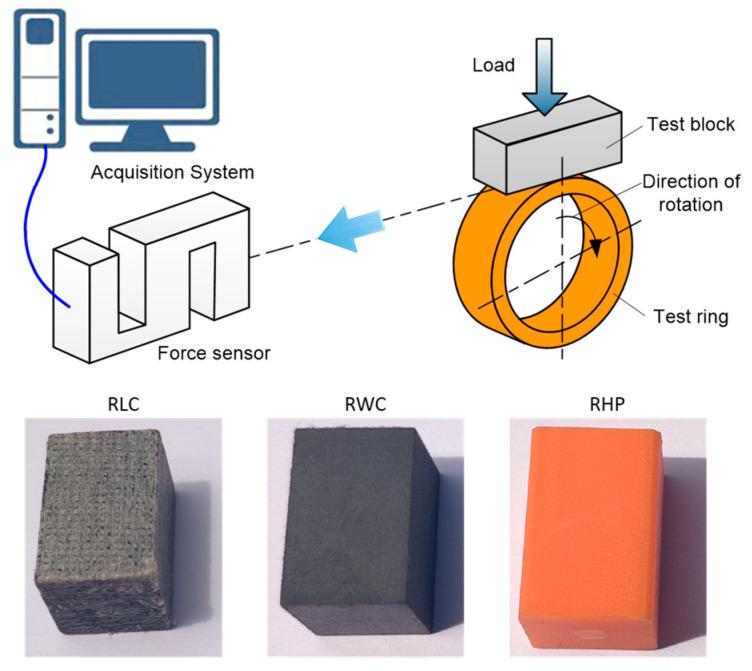
A schematic diagram of the ring on the block tribo-tester and tested blocks.

**Figure 2:**
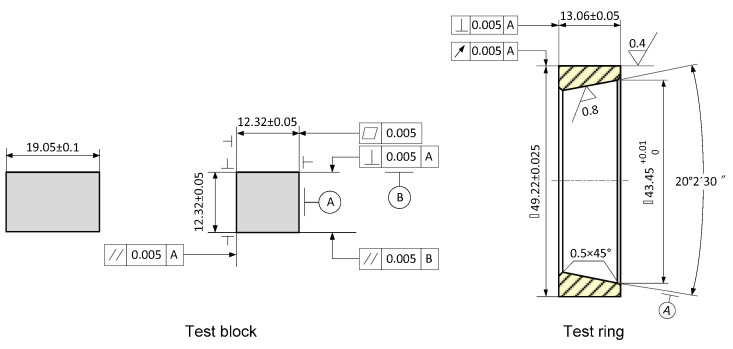
Size of specimen.

To simulate the operation state of the friction pair of water-lubricated bearings, ring block tests were used to research the tribological properties of materials. Test blocks made of three materials were manufactured according to the Chinese national standard GB/T 12444-2006 [27], which specifies the principle and method of the ring block wear test. The colors of the three test blocks are different (as shown in Figure 1), namely gray, black, and orange, respectively. The test block is a standard-sized rectangular block (as shown in Figure 2), and the geometric specification is about 12.32 mm × 12.32 mm × 19.05 mm. The basic physical properties of the composites are shown in Table 1. The compression modulus of RLC and RWC is almost the same. It is about five times the compression modulus of RHP. Although the appearance of RLC looks loose, its compressive strength is the largest. The test ring was made of ZCuSn_10_Zn_2_, and its mechanical properties are shown in Table 2. This material is widely used in marine propulsion shaft sleeves [28]. Its quality inspection needs to be carried out while referencing the Chinese national standard GB/T 1176-2013 [29], which specifies the inspection rules for copper alloys.

### 2.2. Tribological Test

Tribological tests were performed using a commercial ring on a block tribo-tester (MRH-3A, Jinan Yihua Tribology Testing Machine Technology Co., Ltd., Jinan, China [30]). Figure 1 shows the schematics of the test assembly. The maximum load of tested specimens is 3000 N, and the accuracy of the load is ±1% FS. The speed range is 100–3000 rotations/min, and the speed error is ±2% FS. This device converts load (N) into specific pressure (MPa) according to the size of the test block.

During the test, the test block was subjected to a certain load, and it was in contact with the test ring with a determined speed. After a while, the mass loss of the test block was obtained by the weighing method. During the test, the friction and load on the specimens were continuously measured, and the COF was calculated. Test conditions are shown in Table 3. Generally, the marine water-lubricated bearing specific pressure is less than or equal to 0.28 MPa [31]. However, under the propeller eccentric load, the partial pressure may reach 2–3 times the average specific pressure [32]. So, the heavy load conditions are 0.28 MPa, 0.5 MPa, and 0.8 MPa. The linear velocity of the test is in the range of 0.045 m/s–2.24 m/s. This condition covers the low speed and heavy load of marine water-lubricated bearings.

Before each test, the machine operated at a linear velocity of 1.15 m/s for 15 min and then operated under determined conditions for 45 min. Data were recorded after these steps.

Seawater was simulated by adding sodium chloride to distilled water. Regarding the ratio of artificial seawater, 7 g of sodium chloride was weighed using a scale, and 193 g of distilled water was weighed and poured into a 500 mL beaker and stirred until uniform. A 3.5% sodium chloride solution was obtained.

### 2.3. Measurement Techniques and Procedures

Before testing, the wetting angle of specimens was measured using a contact angle measuring instrument (OCA15EC, Dataphysics Instruments, Filderstadt, Germany [33]). The COF was read directly on the ring on the block tribo-tester, and the COF, linear velocity, and load were recorded every 5 s.

The difference in the specimens’ wear quality pre- and post-test was measured using an electronic analytical scale under dry friction conditions and defined as the amount of mass loss under the condition. Under the condition of artificial seawater lubrication, the quality of the specimen was measured using the electronic analytical scale before the test. To eliminate the interference of water absorption after the artificial seawater experiment, the specimens were washed with pure water and then baked in an oven at a temperature of 50 °C for 24 h. After that, the mass was weighed, and the difference between the two masses was the amount of wear loss of specimens under wet conditions [34].

The surface topography of specimens pre- and post-experiment was measured using an ultra-depth three-dimensional microscope (VHX-2000, KEYENCE, Osaka, Japan [35]).

## 3. Results and Discussion

### 3.1. Friction and Wear Properties

**Figure 3:**
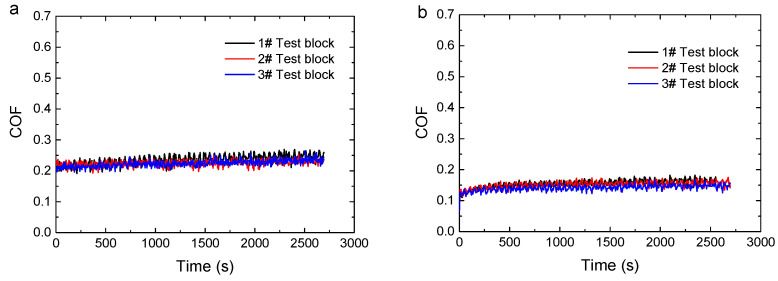
**The** COFs of three test blocks under the same conditions (0.28 MPa; 0.40 m/s): (**a**) dry conditions; (**b**) wet conditions.

Three identical specimens were tested under each condition. The test data were valid when the coincidence degree of the three tests’ data was high and stable (as shown in Figure 3). The COF and mass loss were averaged over three tests.

**Figure 4:**
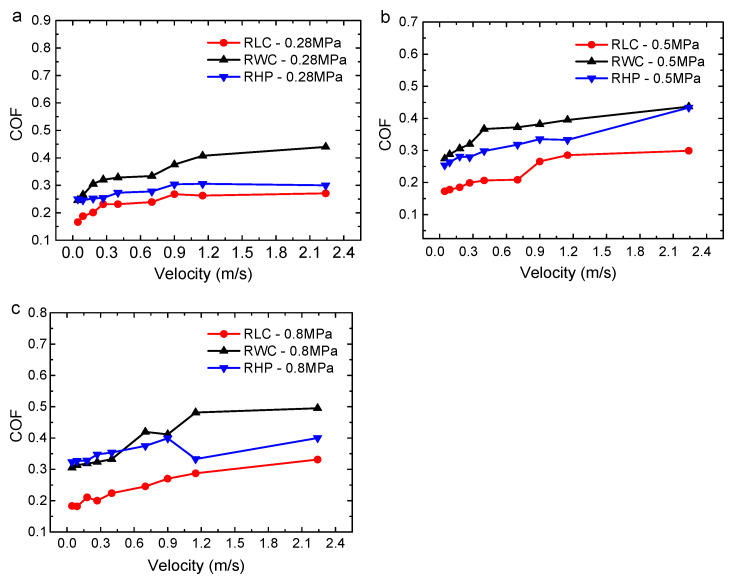
The average COFs of three resin matrix composites with different linear velocities under dry conditions: (**a**) 0.28 MPa; (**b**) 0.5 MPa; and (**c**) 0.8 MPa.

The COFs of the three materials under dry conditions are shown in Figure 4. The COFs of the three materials increase significantly with the increase in velocity. The reason is that the friction pair significantly becomes heated under dry friction, and the temperature increases with the increase in velocity. The fusion of the material causes the contact area and COF to increase.

As shown in Figure 4a, when the specific pressure is 0.28 MPa, the variation in the COF with velocity has an interval characteristic. The linear velocity is between 0 and 0.4 m/s, and the COF increases rapidly with velocity. The linear velocity is between 0.4 m/s and 2.24 m/s, and the COF undergoes little changes with velocity. Then, as the velocity increases, the COF sharply increases. When the specific pressure is 0.5 MPa (as shown in Figure 4b), the linear velocity of 0–0.4 m/s is still the interval for the rapid increase in the COF, and then the COF tends to be stable. When the specific pressure reaches 0.8 MPa (as shown in Figure 4c), the COF of the three materials shows a consistent increasing trend with the increase in velocity. Due to the larger specific pressure, the contact area of the friction pair and the friction heat are large, resulting in the stability of the tribological properties of these materials decreasing, and the tribological properties of the RWC and RHP fluctuate with the change in velocity. As the load increases, the COF also increases at the same linear velocity.

Under the same working conditions, the COF of the RLC is the smallest, and the COF has the smoothest curve with velocity, which indicates that the RLC has the best self-lubricating performance among the three materials. The RWC has the highest COF and the worst self-lubricating properties. The RHP’s self-lubricating properties are between the other two materials.

**Figure 5:**
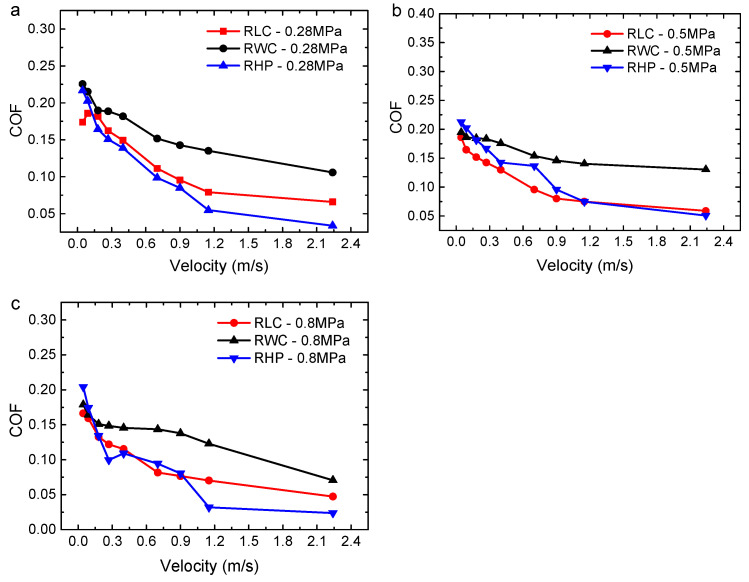
Average COFs of three resin matrix composites with different linear velocities under wet conditions: (**a**) 0.28 MPa; (**b**) 0.5 MPa; and (**c**) 0.8 MPa.

The COFs of the three materials under wet conditions are compared, as shown in Figure 5. The COFs of the three materials decrease with the increase in velocity. When the velocity is between 0.045 m/s and 0.9 m/s, the COF decreases greatly. When the velocity reaches 0.09 m/s, the decreasing tendency of the COF becomes slow and tends to be horizontal. This is because as the velocity increases, more water enters the friction pair, its hydrodynamic effect gradually enhances, and the state of the friction pair gradually changes from dry friction to mixed lubrication, and even to hydrodynamic lubrication. The frictional force of the water film is gradually changed from contact friction to shear friction, so the COF is gradually reduced. When the hydrodynamic region between the friction interfaces reaches the limit, the effect of the water-reducing COF is gradually weakened. It can be predicted that as the velocity continues to rise, the shearing effect of the water film is enhanced, and the COF will increase.

Under the same working conditions, the COFs of the RLC and RHP are small, and the COF of the RWC is the largest. Compared with the RLC, when the load is 0.5 MPa and 0.8 MPa, and the velocity is in the range of 0.40 m/s–1.15 m/s, the COF curve of the RHP fluctuates, indicating that the COF of the RHP in the water state is unstable under a heavy load.

**Figure 6:**
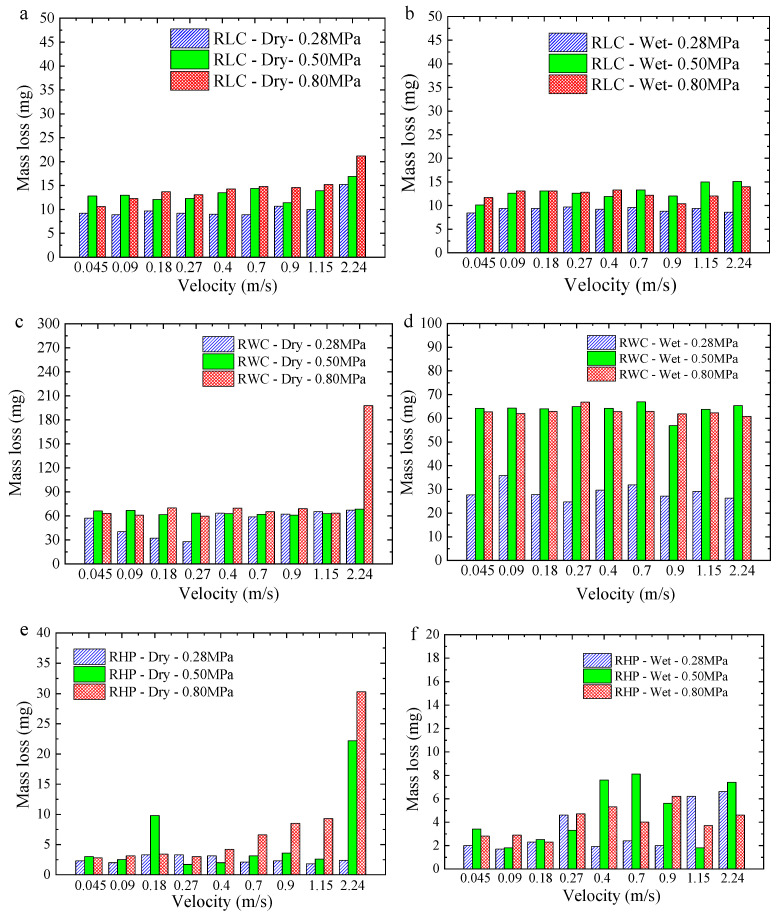
Average mass losses of three resin matrix composites under different conditions: (**a**) RLC—dry; (**b**) RLC—wet; (**c**) RWC—dry; (**d**) RWC—wet; (**e**) RHP—dry; (**f**) RHP—wet.

The mass losses of the three materials under different conditions are shown in Figure 6. As shown in Figure 6a, the mass loss in the RLC gradually increases under dry conditions as the load increases, except for the individual velocity. As the velocity increases, the mass loss of the RLC also increases, but the amplitude is not obvious. Compared to the dry condition, there is a small reduction in the mass loss under wet conditions (as shown in Figure 6b). Overall, the RLC mass loss is low and steady as the operating conditions change. This indicates that the RLC has very good self-lubricating and water-lubricating properties.

As shown in Figure 6c, under dry conditions, the mass loss of the RWC is large, about 60 mg, and when the velocity is 2.24 m/s and the load is 0.80 MPa, the mass loss of the RWC sharply increases to about 198 mg, and the wear is serious. Under wet conditions (as shown in Figure 6d), when the load is 0.28 MPa, the mass loss is about 30 mg, which is significantly lower than that in dry conditions. When the load is 0.5 MPa and 0.8 MPa, mass losses at different velocities are close, about 65 mg. The phenomenon of a sharp increase in wear loss does not appear again, indicating that wet conditions improve the lubrication state of the friction pair. The wear loss of the RHP is the smallest among the three materials, followed by the RLC, with the RWC experiencing the largest wear loss.

### 3.2. Observation and Analysis of Surface Topography

**Figure 7:**
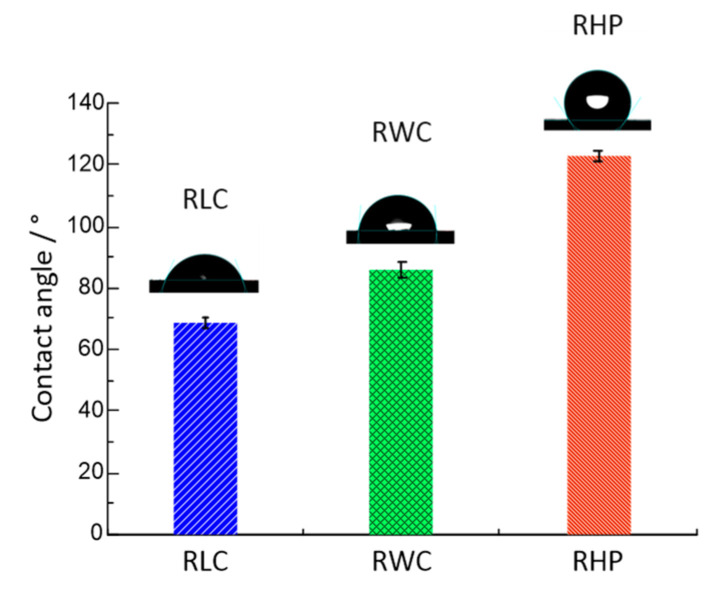
Wetting angles of three test blocks.

Figure 7 illustrates the wetting angles of the resin matrix composites. The wetting angle of the RHP was the highest at 123 ± 2°. The wetting contact angles of the RLC and RWC were 68 ± 2.5° and 86 ± 4.5°, respectively. The analysis indicates that the RLC exhibits superior wettability, whereas wetting the RHP surface is challenging.

**Figure 8:**
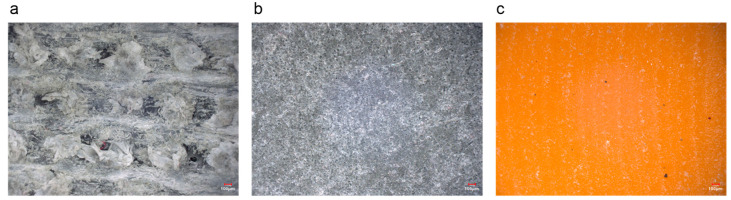
Surface topography of three resin matrix composites before testing: (**a**) RLC; (**b**) RWC; and (**c**) RHP.

Figure 8 depicts the surface topography of the resin matrix composites before testing. The RLC surface exhibits a reticulum texture with floccule fibers (as depicted in Figure 8a). In contrast, the surface of the RWC (illustrated in Figure 8b) is dense, with graphite and MoS_2_ particles dispersed and embedded within the material. The RHP (shown in Figure 8c) displays a smooth, dense surface with visible scratches and residual agglomerated resin post-machining.

**Figure 9:**
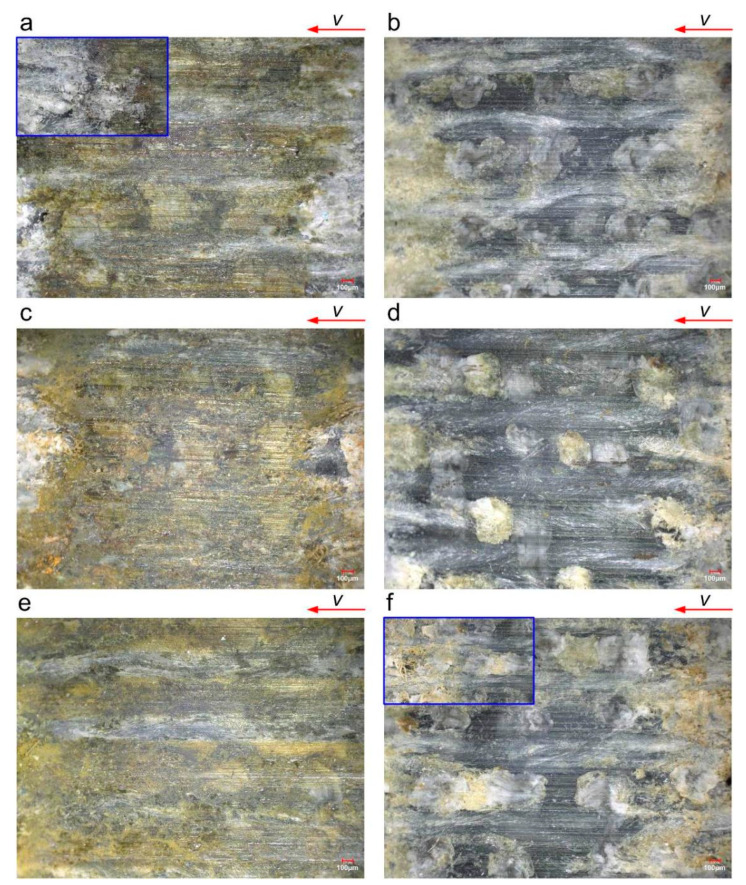
Surface topography of RLC after testing (0.5 MPa): (**a**) dry—0.045 m/s; (**b**) wet—0.045 m/s; (**c**) dry—0.27 m/s; (**d**) wet—0.27 m/s; (**e**) dry—0.9 m/s; (**f**) wet—0.9 m/s. The blue box highlights flocculation and bare material texture after rubbing.

Figure 9 shows the surface topography of the RLC after testing. The test conditions are as follows: 0.5 MPa; dry and wet; 0.045 m/s, 0.27 m/s, and 0.9 m/s. Figure 9a,c,e show images taken under dry conditions. Figure 9b,d,f show images taken under wet conditions. Under dry conditions, a large area of brownish yellow appears on the surface of the specimen, and only a small area of brownish yellow appears under wet conditions. Under dry conditions, the frictional heat of the material is greater than that under wet conditions, and a high temperature causes the material to melt and turn yellow. It can also be seen from Figure 9a,c that the floc is curled and yellowed, and the floc is deposited on one side of the wear scar. The width of the worn area under dry conditions is greater than that under wet conditions. Under wet conditions and a velocity of 0.9 m/s, the worn area is smaller than that under the other working conditions. This is because the friction interface enters more water at a high speed, reduces the direct contact area, and reduces wear loss. Under wet conditions, the reticulum texture of the surface is worn to reveal the next layer of texture.

**Figure 10:**
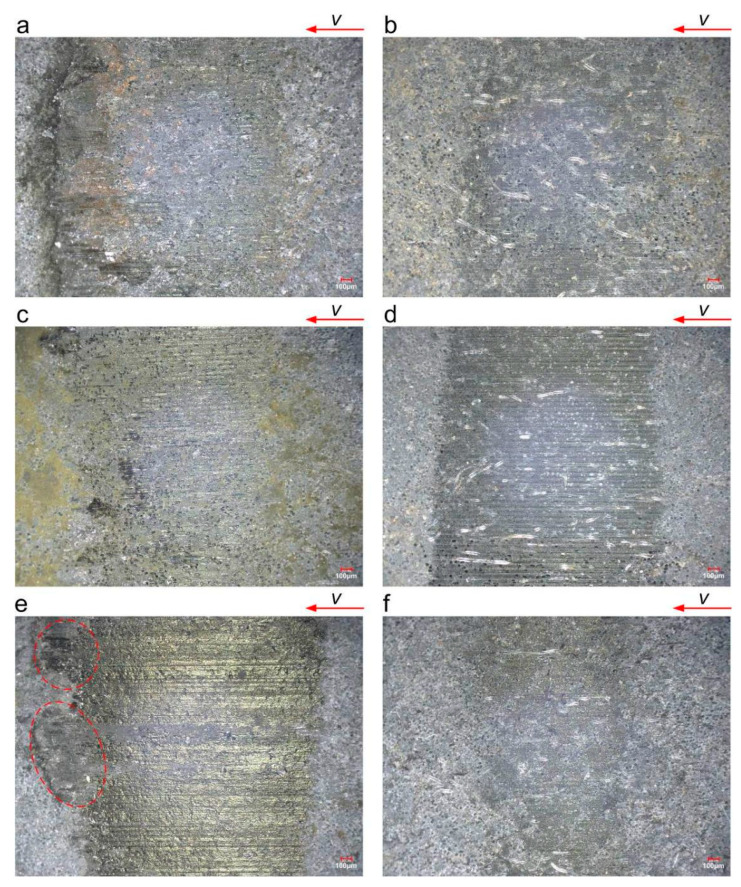
Surface topography of RWC after testing (0.5 MPa): (**a**) dry—0.045 m/s; (**b**) wet—0.045 m/s; (**c**) dry—0.27 m/s; (**d**) wet –0.27 m/s; (**e**) dry—0.9 m/s; (**f**) wet –0.9 m/s. The red dashed box shows the wear products that accumulate at the edge of the friction area.

Figure 10 shows the surface topography of the RWC after testing. The test condition was the same as that used for the RLC. Under dry conditions, the friction area also appears brownish yellow, and the material is burnt and yellow due to the high temperature of dry friction. However, the yellow area is smaller than that of the RLC. Under dry conditions and a velocity of 0.9 m/s, the friction area is completely melted and brightened, and the wear product accumulates on the side of the wear scar, indicating that the wear and heat phenomenon is serious. Under wet conditions, water improves the wear state of the friction pair significantly, and no yellow area appears. Under wet conditions and a velocity of 0.9 m/s, the worn area is significantly smaller than that under other conditions. The friction area is smooth and has horizontal scratches under both dry and wet conditions.

**Figure 11:**
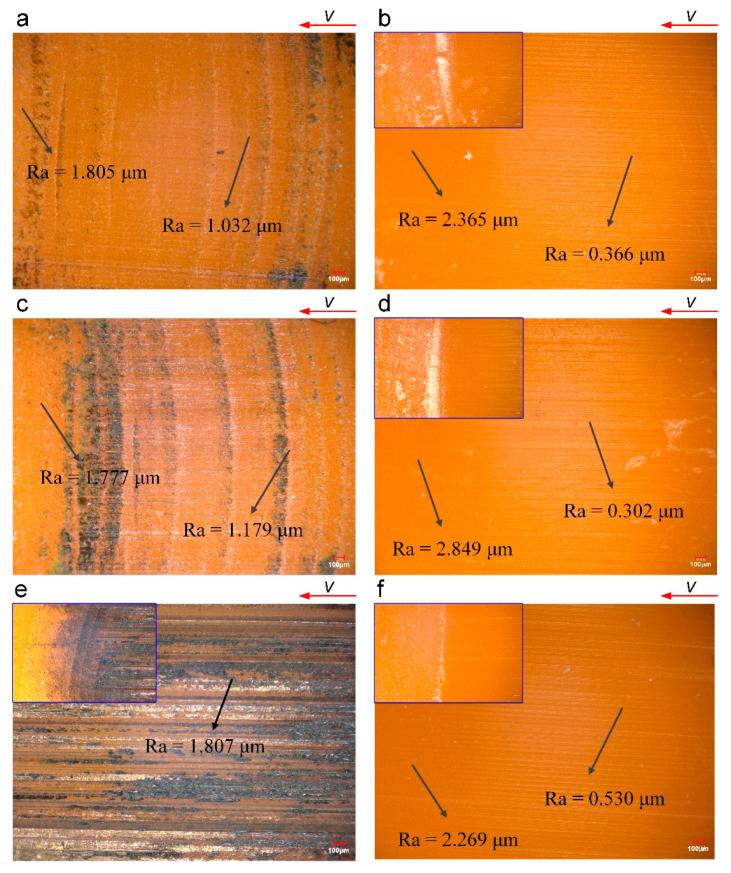
Surface topography of RHP after testing (0.5 MPa): (**a**) dry—0.045 m/s; (**b**) wet—0.045 m/s; (**c**) dry—0.27 m/s; (**d**) wet—0.27 m/s; (**e**) dry—0.9 m/s; (**f**) wet –0.9 m/s. The blue box is enlarged to show the worn products after friction. (**e**) shows the worn products burnt and blackened, while those in (**b**,**d**,**f**) are white.

Figure 11 shows the surface topography of the RHP after testing. Unlike the RLC and RWC, under dry conditions, black wear products appear in the worn area of the RHP, and as the velocity increases, the black product increases significantly. At a velocity of 0.9 m/s, the worn area is all burnt and black. This indicates that the RHP has poor heat resistance. Under dry conditions, besides horizontal wear scars, the surface also shows vertical wear scars at velocities of 0.045 m/s and 0.27 m/s. This vertical scar is initially caused by machining (as shown in Figure 8c). During the contact friction process, the surface bulge has a high friction temperature, and the burnt deposit accumulates in the groove to deepen the display of the vertical wear scar. As the velocity keeps increasing, large areas of wear and scorching appear on the surface, and vertical wear scars disappear. Under wet conditions, water takes friction heat, and the high-temperature blackening phenomenon does not occur. The friction surface only has tiny horizontal scratches and has a polishing effect on the vertical processing texture. So, the RHP has good water lubrication characteristics. As shown in Figure 11b,d,f, a white agglomerate of wear product appears at the wear edge.

**Figure 12:**
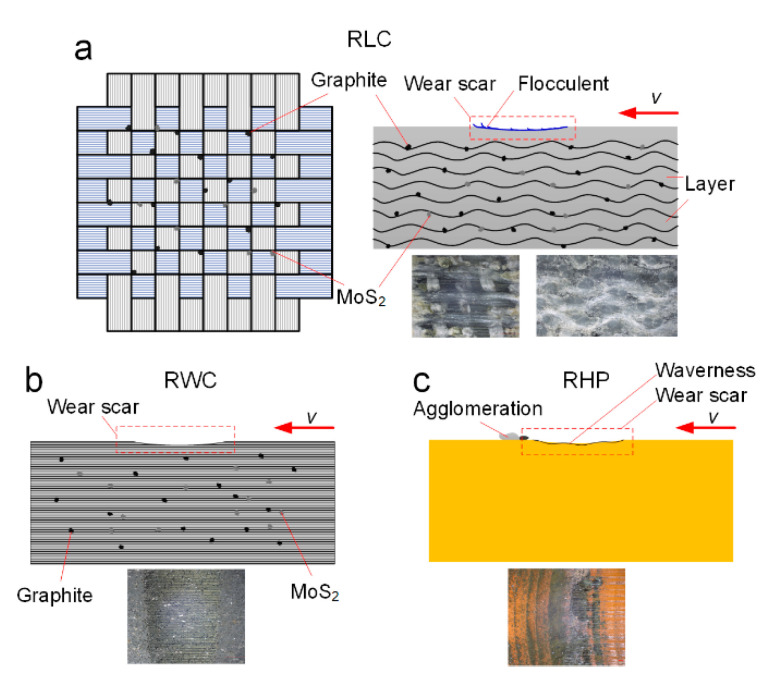
Wear characteristics of three resin matrix composites: (**a**) RLC; (**b**) RWC; and (**c**) RHP.

The wear mechanism of the three materials was summarized by analyzing the friction and wear characteristics of the three materials (as shown in Figure 12).

An RLC is a fiber-reinforced polyester/resin-based laminated composite, and the single layer is formed into a netlike layer, and a lamination forming process (as shown in Figure 12a) is adopted between the layers. There is wave layering at the side of the specimen. Graphite and MoS_2_ particles are dispersed between the layers. The surface has a “loose” reticulum texture and floccule fibers, contributing to heat dissipation and improvements in material wettability and water storage capacity. Following wear, certain fibers become broken. Due to the pull force of the fiber web, the broken fibers are not easily torn off, and the space for storing water increases. At the same time, the exposed graphite and MoS_2_ particles can also be used for self-lubricating.

An RWC is a fiber-reinforced resin-based winding composite obtained using a filament winding process (as shown in Figure 12b). The surface is dense, and graphite and MoS_2_ particles are embedded in the material. The dense surface is not conducive to heat dissipation and the storage of lubricating water. After abrasion, the fibers are cut, and the exposed graphite and MoS_2_ particles may fall off. This may be why the tribological performance of the RWC is relatively poor.

An RHP is a thermosetting resin-based homogeneous polymer and is a non-metal polymer compound (as shown in Figure 12c). The surface of this polymer material is smooth and dense, which exhibits hydrophobic properties. The material has good tribological properties under water lubrication. However, its temperature resistance is poor under dry conditions, making it prone to the burning and melting of large areas. The material exhibits agglomerate wear and removal. Water-lubricated bearings of ships have poor working conditions, and there are inevitable contact and dry friction conditions. The wear resistance and wear characteristics of the RHP are not suitable for dry friction.

## 4. Conclusions

The wear behaviors of the RLC, RWC, and RHP blocks and ZCuSn_10_Zn_2_ ring rubbing pairs under simulated environment conditions on a ring-on-block wear tester were investigated. The main conclusions of this work are summarized below:

(1) The COFs of the three materials increase significantly with the speed or load under dry conditions. The COF of the RLC is the smallest and has the best self-lubrication. Under wet conditions, the COFs of the three materials decrease with the increase in speed or load and show an obvious hydrodynamic effect. The COF and mass loss of the RWC are the largest, and the RLC and RHP have small COFs and mass loss values.
(2) The surface of the RLC has a reticulum texture. The friction area is brownish and yellow under dry conditions, and a curled floccule appears. The surface of the RWC is dense, and the friction area under dry conditions is melted and brightened. The surface of the RHP is smooth, the worn product has an agglomerate shape, and the surface is easily burnt and blackened under dry friction.
(3) In the process of wear evolution, the laminated forming method has a reticulum texture and floccule fibers. This structure is good for heat diffusion, material wettability, and water storage capacity. Wear-fractured fibers increase the space for storing water and facilitate the release of self-lubricating materials. This forming method has better tribological performance.
(4) In this paper, the tribological properties of various materials were evaluated using a ring block testing machine under a range of operating conditions. Subsequently, the same testing program and equipment can be employed to investigate other types of composites, including ultrahigh-molecular-weight polyethylene (UHMWPE), polytetrafluoroethylene (PTFE), thermoplastic polyurethane etc.

## Figures and Tables

**Table 1:** Properties of resin matrix composites (at room temperature, 25 °C).

Physical Properties	RLC	RWC	RHP
Compression modulus (MPa)	2320	2300	440
Compressive strength (MPa)	280	80	30
Density (g/cm^3^)	1.30	1.34	1.16
Hardness	90 (HRM)	70 (HRM)	67 (shore D)
Thermal expansion coefficient (×10^−5^ °C^−1^)	9	6	15.1–21.1
Water absorption coefficient (%)	0.1	0.2	1.3

**Table 2:** Main element proportions of ZCuSn_10_Zn_2_.

Tensile Strength (MPa)	Yield Strength	Elongation	Hardness (HRM)
270	140	7	80

**Table 3:** List of test conditions ^a^.

No.	Lubrication Condition	Load (MPa)	Linear Velocity (m/s)
1	Dry Wet ^b^	0.28 0.5 0.8	0.045
2			0.09
3			0.18
4			0.27
5			0.40
6			0.77
7			0.90
8			1.15
9			2.24

^a^ 2 (wet/dry) × 3 (load) × 9 (speed). ^b^ Sea water lubrication.

## Data Availability

Data is contained within the article.
